# The Long Pentraxin PTX3 Is an Endogenous Inhibitor of Hyperoxaluria-Related Nephrocalcinosis and Chronic Kidney Disease

**DOI:** 10.3389/fimmu.2018.02173

**Published:** 2018-09-25

**Authors:** Julian A. Marschner, Shrikant R. Mulay, Stefanie Steiger, Lidia Anguiano, Zhibo Zhao, Peter Boor, Khosrow Rahimi, Antonio Inforzato, Cecilia Garlanda, Alberto Mantovani, Hans-Joachim Anders

**Affiliations:** ^1^Nephrologisches Zentrum, Medizinische Klinik und Poliklinik IV, Klinikum der Universität München, Munich, Germany; ^2^Department of Nephrology, Institute of Pathology, RWTH University of Aachen, Aachen, Germany; ^3^DWI-Leibniz Institute for Interactive Materials, Aachen, Germany; ^4^Department of Inflammation and Immunology, Humanitas Clinical and Research Center, Rozzano, Italy; ^5^Department of Biomedical Sciences, Humanitas University, Pieve Emanuele, Italy

**Keywords:** kidney stone, colic, hyperoxaluria, crystals, nephrolithiasis, urolithiasis, PTX3

## Abstract

The long pentraxin 3 (PTX3) exerts a variety of regulatory functions in acute and chronic tissue inflammation. In particular, PTX3 acts as an opsonin for a variety of pathogens and endogenous particles. We hypothesized that PTX3 would exhibit opsonin-like functions toward calcium oxalate crystals, too, and inhibit crystal growth. This process is fundamental in kidney stone disease as well as in hyperoxaluria-related nephrocalcinosis, the paradigmatic cause of chronic kidney disease (CKD) in children with primary hyperoxaluria type I due to genetic defects in oxalate metabolism. Direct effects of PTX3 on calcium oxalate crystals were investigated *in chemico* by adding recombinant PTX3 to supersaturated calcium and oxalate solutions. PTX3, but not isomolar concentrations of albumin, dose-dependently inhibited crystal growth. *In vivo*, the PTX3 protein was undetectable in tubular epithelial cells and urine of wild-type mice under physiological conditions. However, its levels increased within 3 weeks of feeding an oxalate-rich diet, an exposure inducing hyperoxaluria-related nephrocalcinosis and CKD in selected mouse strains (male and female C57BL/6N and male Balb/c mice) but not in others (male and female 129SV and CD-1, male and female Balb/c mice). Genetic ablation of *ptx3* in nephrocalcinosis un-susceptible B6;129 mice was sufficient to raise the oxalate nephropathy phenotype observed in susceptible strains. We conclude that PTX3 is an endogenous inhibitor of calcium oxalate crystal growth. This mechanism limits hyperoxaluria-related nephrocalcinosis, e.g., in primary or secondary hyperoxaluria, and potentially also in the more prevalent kidney stone disease.

## Introduction

Pentraxins are immunoregulatory acute phase proteins induced upon disruption of homeostasis (1). The short pentraxins, C-reactive protein and serum amyloid P are synthesized in hepatocytes in response to IL-6. C-reactive protein and serum amyloid P act as opsonins binding to a number of microorganisms, dead cells, and other particles to facilitate complement–mediated killing or phagocytosis (2). As opposed to the short pentraxins, the long pentraxin PTX3 is locally produced and released at sides of infection and inflammation by several immune and non-immune cells, including neutrophils, macrophages, myeloid dendritic, endothelial and epithelial cells in response to inflammatory cytokines (i.e., IL-1β and TNF-α) and Toll-like receptor agonists (3, 4). Importantly, expression of PTX3 has been documented in the murine uroepithelium during uropathogenic *E. coli* (UPEC) infections, where it enhances phagocytosis of UPEC by innate immune cells (5). Indeed, amongst the numerous immunoregulatory functions of this pentraxin, recognition of extracellular particles (i.e., microbial moieties) and promotion of their phagocytosis by macrophages, neutrophils and dendritic cells are fundamental opsonic activities (6–10).

Some extracellular particles are of crystalline nature and account for a broad spectrum of acute and chronic diseases (11). Numerous studies show that the cellular arm of the immune system handles crystalline and non-crystalline extracellular particles in a similar way, however little is known regarding the role of humoral immune elements in the recognition and control of crystalline particles (12–14). Here we focus on the interaction of PTX3 with calcium oxalate (CaOx) crystals. CaOx stones account for the vast majority of calculi in kidney stone disease, i.e., nephro- and urolithiasis, affecting around 12% of men and 5% of women during their lifetime (15). In addition, intrarenal CaOx crystal retention causes nephrocalcinosis, a state that is usually asymptomatic but can lead to progressive nephron loss and chronic kidney disease (CKD), especially in rare genetic forms of hyperoxaluria (15, 16). The traditional pathogenic concept of nephrolithiasis and nephrocalcinosis is based on urine supersaturation of minerals or on the lack of sufficient crystallization inhibitors (17–19). Intratubular microcrystals adhere to the luminal membrane of tubular epithelial cells via a group of adhesion molecules (20–28). Adherent microcrystals grow by apposition of minerals and ultimately form crystal plugs obstructing tubules followed by nephron atrophy, interstitial inflammation and fibrosis with loss of renal excretory function, i.e., CKD (16, 29, 30).

Serum proteins, such as albumin as well as plasma fractions containing alpha-globulins and beta-globulins inhibit CaOx crystal aggregation via a variety of mechanisms (31, 32). We recently observed that also the humoral immune effector and opsonin immunoglobulin G inhibits CaOx crystal growth *in vitro* (33). Because of their high molecular weight neither albumin nor IgG pass the filtration barrier and are therefore not constituents of the glomerular ultrafiltrate or urine in healthy individuals and stone formers. We therefore hypothesized that an opsonin, such as PTX3, which is likely expressed by tubular epithelial cells (i.e., beyond the renal filtration barrier) and, therefore, directly released into the urine (5), may act as an endogenous inhibitor of CaOx crystal aggregation inside renal tubules. Thereby PTX3 might limit nephrocalcinosis during hyperoxaluria, a hypothesis that is supported by the evidence presented and discussed in this study.

## Results

### Recombinant human PTX3 inhibits supersaturation-induced CaOx crystal aggregation

**Figure 1:**
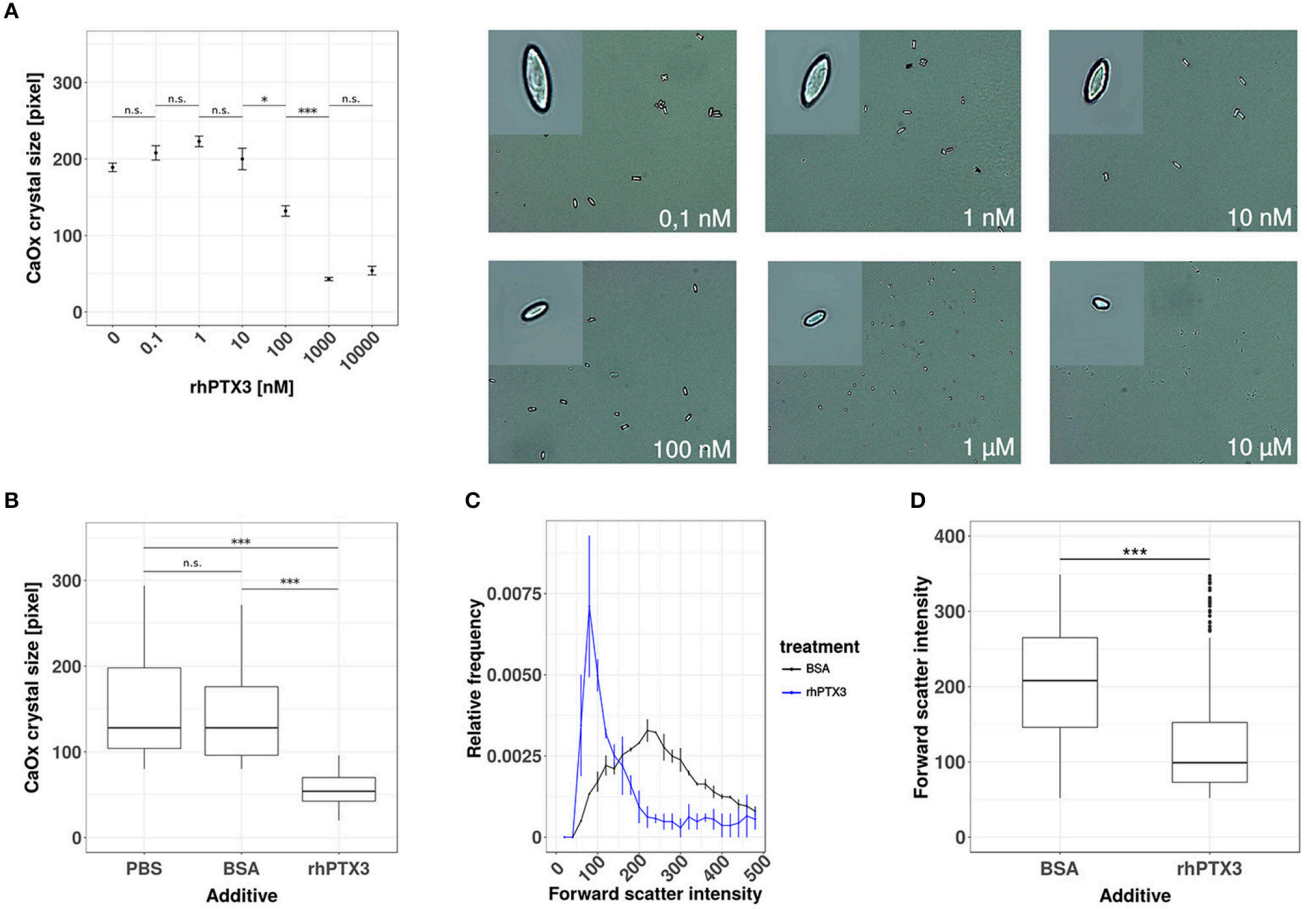
Recombinant human PTX3 inhibits supersaturation-induced CaOx crystal aggregation. **(A)** Calcium oxalate crystals were generated *in chemico* in the presence of increasing concentrations of rhPTX3 and crystal size was measured microscopically 24 h later. **(B)** The effect of 1 μM rhPTX3 on microscopically measured calcium oxalate crystal size was compared to equimolar bovine serum albumin (BSA) and PBS. **(C,D)** Calcium oxalate crystals generated in the presence of 1 μM BSA or equimolar rhPTX3 were compared for their forward scatter (FSC) signal intensity by flow cytometry and displayed for **(C)** relative frequency of FSC and **(D)** overall FSC intensity. Data are from three independent experiments and represent means ± SEM in **(A)**. n.s., not significant; *p < 0.05, ***p < 0.001 between groups as indicated.

To test our hypothesis, we first added increasing doses of recombinant human PTX3 or equimolar concentrations of bovine serum albumin (BSA) to a supersaturated solution of sodium oxalate and calcium chloride. The crystal preparation was optimized to produce calcium oxalate monohydrate crystals, since this species is mostly found in human oxalate kidney stones (34, 35). Calcium oxalate monohydrate crystal formation was confirmed by phase contrast microscopy and the crystal size was assessed by digital morphometry 24 h later. PTX3, but not bovine serum albumin, dose-dependently reduced crystal size statistically significantly from a concentration of 100 nM or higher (Figures 1A,B). The effect of rhPTX3 on calcium oxalate crystal size distribution and, for comparison, that of equimolar concentrations of BSA were confirmed by flow cytometry based on intensity of the forward scatter signal from CaOx monohydrate crystals (Figures 1C,D). Noteworthy, the reported inhibitory effects of human albumin on kidney stone formation rely on a different mechanism, which favors generation of di- and trihydrate forms of calcium oxalate crystals (36). Both types of crystals adhere poorly to the luminal membrane of tubular epithelial cells and are flushed out more easily (25). Hence we conclude that the long pentraxin PTX3 inhibits aggregation and growth of CaOx monohydrate crystals *in vitro* at nanomolar concentrations in a dose-dependent manner.

### Hyperoxaluria-induced nephrocalcinosis is associated with tubular PTX3 secretion

**Figure 2:**
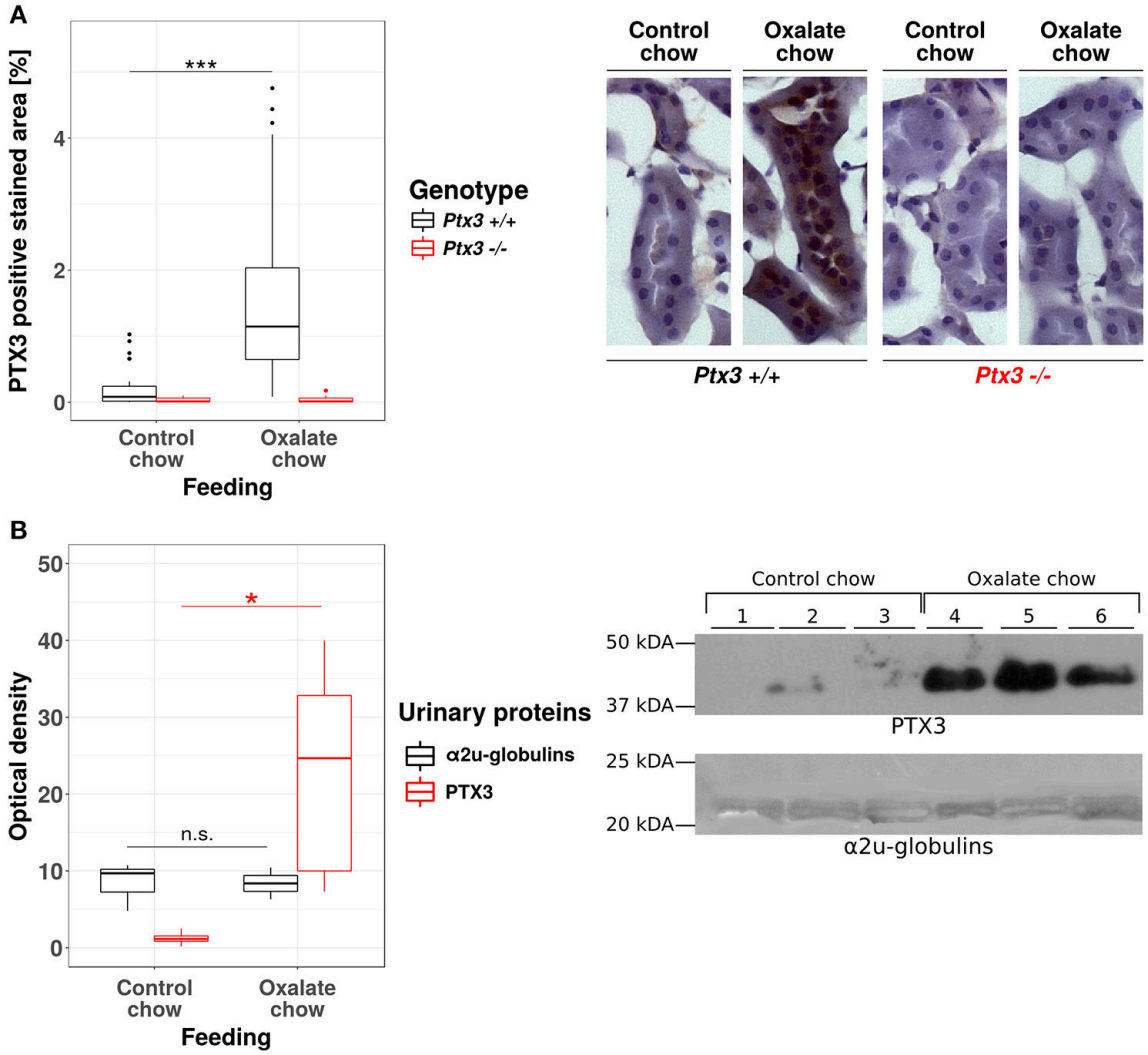
Hyperoxaluria-induced nephrocalcinosis is associated with tubular PTX3 secretion. Female PTX3-competent (*Ptx3*^+/+^) and -deficient (*Ptx3*^−/−^) animals with 10–12 weeks of age received either control or oxalate chow for 3 weeks (*n* = 5). **(A)** Tubular expression of PTX3 was analyzed by immunohistochemical staining in frozen kidney sections and quantified as positive stained area per section in percent. **(B)** Detection of PTX3 in whole urine samples by immunoblotting (25 μg protein/lane, upper right). Ponceau Red staining shows major urinary proteins (mainly a2u-globulins, ~20 kDa, lower right) as a loading control. Optical density of the immunoreactive and Ponceau Red stained bands was quantified by ImageJ (NIH, Bethesda, MD). Data are from two independent experiments. n.s., not significant; *p < 0.05, ***p < 0.001 between groups as indicated.

The human PTX3 is a 381 amino acid glycoprotein with a predicted molecular weight of 40.165 Da. However, the recombinant protein runs in SDS-PAGE gels at an apparent MW of ~45 kDa, due to N-linked glycosylation at Asn220 (37, 38). The functional PTX3 molecule is an homo-oligomer that comprises eight identical protomer subunits (i.e., octamer) with a total MW of ~340 kDa (39, 40), which would most likely prevent circulating PTX3 from passing the glomerular filtration barrier and reaching the urine (40). PTX3 is expressed by the murine uroepithelium during UPEC infections, and has been described in the bladder of patients with urinary tract infections (UTIs) (5), however expression of this pentraxin in the human uroepithelium has not been documented yet. Therefore, before assessing the role of PTX3 on CaOx crystal growth *in vivo*, we first examined whether renal epithelial cells express PTX3 and excrete it into the urine. The human protein atlas (41, 42) reports no baseline expression of PTX3 mRNA in four human renal epithelial cell lines (HEK293, NTERA-2, PC-3, RPTEC-TERT1, data available at https://www.proteinatlas.org/ENSG00000163661-PTX3/cell), no epithelial PTX3 positivity using immunohistochemistry in healthy kidney samples from three different donors (data available at https://www.proteinatlas.org/ENSG00000163661-PTX3/tissue/kidney#imid_18912581) and reports negative staining results in 12 renal cell carcinoma samples (data available at https://www.proteinatlas.org/ENSG00000163661-PTX3/ pathology). Consistent with this scenario in humans and previous findings in mice (5), no staining was observed for PTX3 in the kidney of normal mice. However, a remarkable induction of tubular PTX3 expression was apparent upon feeding mice an oxalate-rich diet (Figure 2A). Specificity of the immunostaining was validated using an identical staining protocol on renal sections of *Ptx3-*deficient mice (Figure 2A). Consistent with these findings, western blot analyses of urine samples from healthy mice on normal chow diet could not detect the PTX3 protein, whereas those performed on mice with diet-induced hyperoxaluria clearly showed it in the urine (Figure 2B). Thus, renal tubular cells have the capacity to express PTX3 and secrete it into the urine, e.g., during hyperoxaluria.

### Lack of PTX3 induces hyperoxaluria-induced nephrocalcinosis in non-susceptible B6;129SV mice

**Figure 3:**
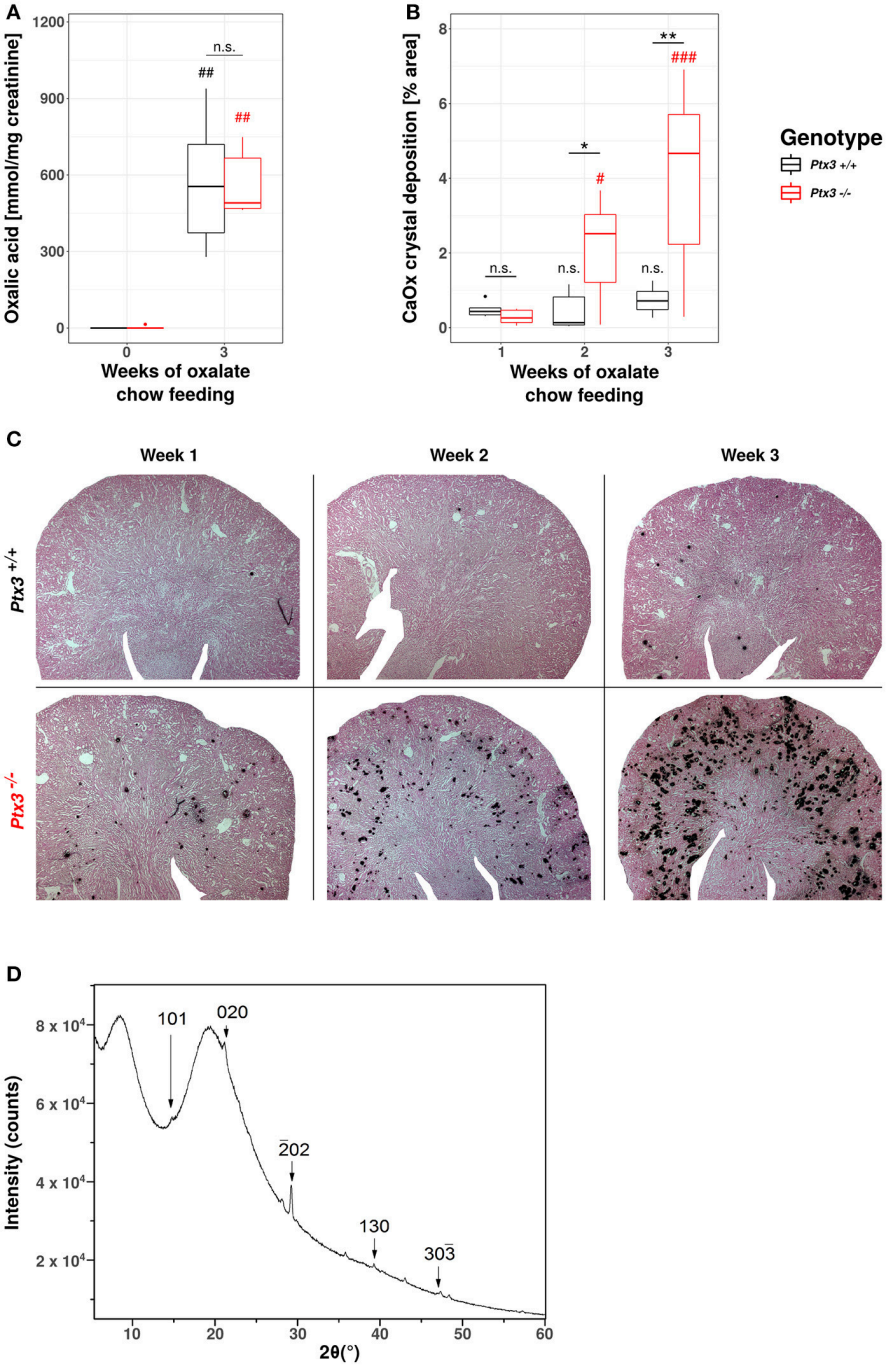
Lack of PTX3 induces hyperoxaluria-induced nephrocalcinosis in non-susceptible B6;129SV mice. Female PTX3-competent (*Ptx3*^+/+^) and -deficient (*Ptx3*^−/−^) animals with 10–12 weeks of age received oxalate chow for 3 weeks (*n* = 9). **(A)** Oxalic acid levels in urine at baseline and after 3 weeks of oxalate chow. **(B)** Quantification of calcium oxalate crystal deposition was conducted using Pizzolato's method on formalin fixed kidney sections as shown in **(C)**. **(D)** Analysis of *PTX3*^−/−^ kidneys by X-ray diffraction consistent with calcium-oxalate monohydrate crystals. Data are from four independent experiments. n.s., not significant; *p < 0.05, ** *p*< 0.01 vs. wild type and respective #, ##, and ### vs. week 1 as indicated.

Having shown that PTX3 is present in the urine during hyperoxaluria, we sought to design an experiment that could examine its putative role as an inhibitor of CaOx crystallization. We selected the B6;129-Ptx3^tm1Mant^ mice that has global PTX3-deficiency to unravel the role of this pentraxin in CaOx crystal aggregation-related disease, i.e., in a model resembling the kidney phenotype of primary hyperoxaluria type I or other forms of progressive nephrocalcinosis. *Ptx3*^−/−^ or *Ptx3*^+/+^ littermates from heterozygous breeders were fed with a sodium oxalate-rich diet for 3 weeks. Despite the equal induction of hyperoxaluria in both genotypes (Figure 3A), only the *Ptx3*-deficient mice developed a nephrocalcinosis (Figures 3B,C). X-ray diffraction on pulverized and freeze dried murine *Ptx3*^−/−^ kidney tissue, that were fed for 3 weeks with oxalate chow, confirmed the presence of calcium oxalate monohydrate crystals in the kidneys, as judged based on the diffraction angle pattern (Figure 3D). Therefore, the B6;129 mice were not susceptible *per se* to hyperoxaluria-induced nephrocalcinosis, however deletion of the *ptx3* gene was sufficient to induce nephrocalcinosis in these animals, thus rendering them similar to other strains that are prone to nephrocalcinosis (43).

### Lack of PTX3 induces hyperoxaluria-induced progressive CKD in non-susceptible B6;129 mice

**Figure 4:**
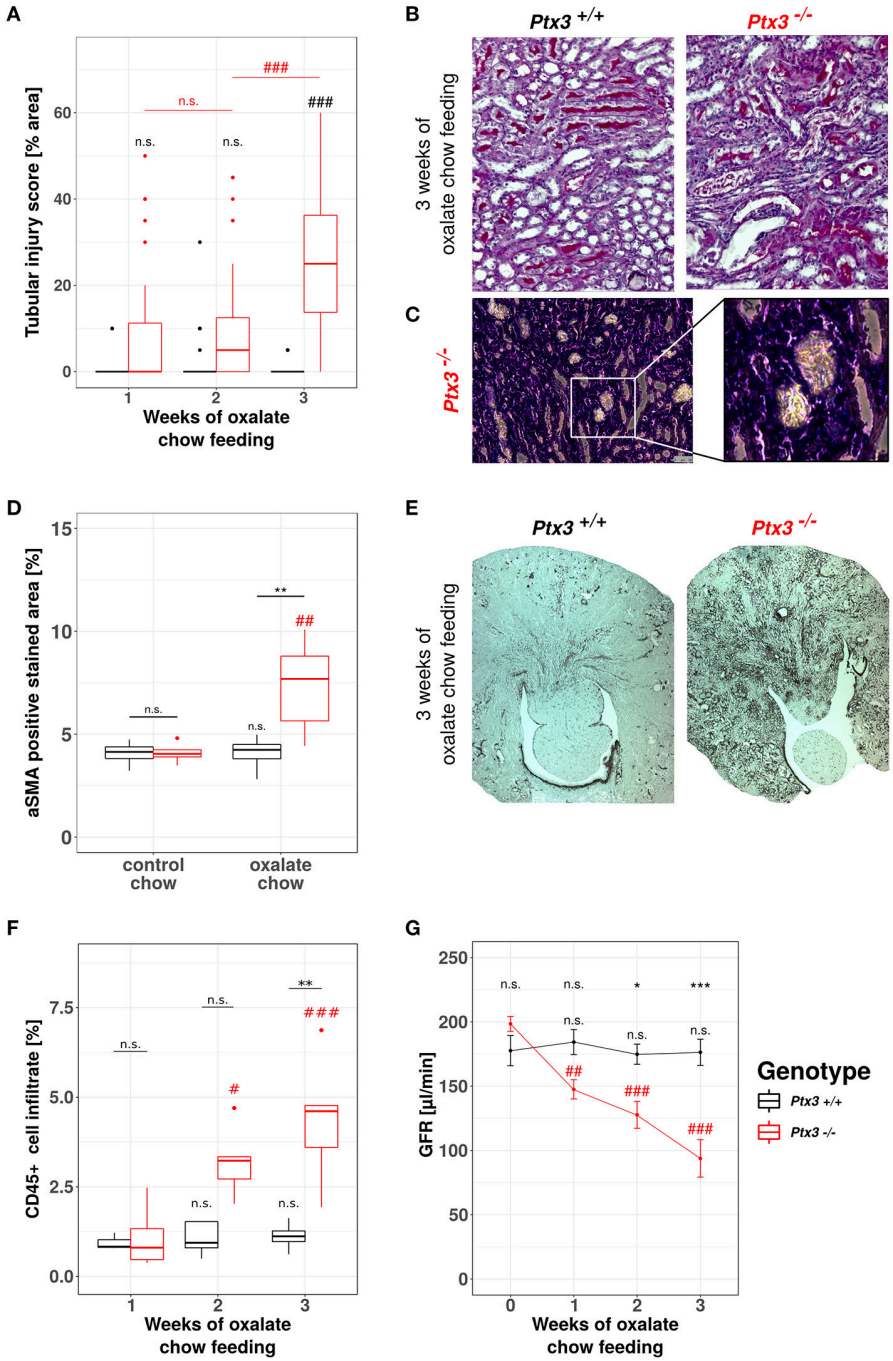
Lack of PTX3 induces hyperoxaluria-induced progressive CKD in non-susceptible B6;129 mice. *Ptx3*^+/+^ and *Ptx3*^−/−^ female littermates (10–12 weeks of age) were fed with oxalate chow for 3 weeks (*n* = 9). **(A)** Quantification of tubular injury was conducted using PAS-stained kidney sections as shown in **(B)**. **(C)** Phase contrast image of crystal plugs in H&E-stained kidney section in 200× (left) and 400× (right) magnification. **(D)** Quantification of αSMA-positive area in kidney sections after 3 weeks of either control or oxalate chow feeding as shown in **(E)**. **(F)** Percentage of CD45^+^ cells within whole kidney cell suspension assessed by flow cytometry. **(G)** GFR assessed by transcutaneous measurement of FITC-sinistrin from subcutaneous capillaries at weeks 0, 1, 2, and 3 after onset of high oxalate diet in both genotypes. Data are from four independent experiments and represent means ± SEM in **(G)**. n.s., not significant; *p < 0.05, **p < 0.01, ***p < 0.001 vs. wild type and respective #, ##, and ### vs. week 1 as indicated.

A detailed phenotype analysis revealed profound and progressive nephrocalcinosis-related tubular injury in Periodic acid-Schiff (PAS)-stained kidney sections (Figures 4A,B) with CaOx crystal plugs (Figure 4C) in dilated tubules in *Ptx3*^−/−^ but not in *Ptx3*^+/+^ B6;129 mice. Tissue remodeling was further obvious from diffuse cortical and medullary interstitial fibrosis as indicated by interstitial positivity for smooth muscle actin (Figures 4D,E). Diffuse interstitial leukocyte infiltrates as assessed by flow cytometry from whole kidney homogenates (Figure 4F) indicated ongoing inflammatory processes. Finally, renal excretory function was assessed by measuring glomerular filtration rate (GFR) in awake and unrestrained mice using FITC-sinistrin injection and quantifying vascular FITC clearance kinetics with a transdermal detector (Figure 4G). *Ptx3*^−/−^ and *Ptx3*^+/+^ mice showed identical GFR before onset of oxalate diet feeding. While GFR remained constant in *Ptx3*^+/+^ mice over the entire feeding period, *Ptx3*^−/−^ mice showed a linear decline of GFR as previously reported for susceptible mouse strains (43). Thus, *Ptx3*-deficient mice display the full phenotype of hyperoxaluria-induced nephrocalcinosis and progressive CKD. Together with the *in vitro* data described above, we conclude that PTX3 is an endogenous inhibitor of CaOx crystal aggregation, nephrocalcinosis and CKD during hyperoxaluria.

### PTX3 abolishes crystal adhesion molecule expression *in vitro* and *in vivo*

**Figure 5:**
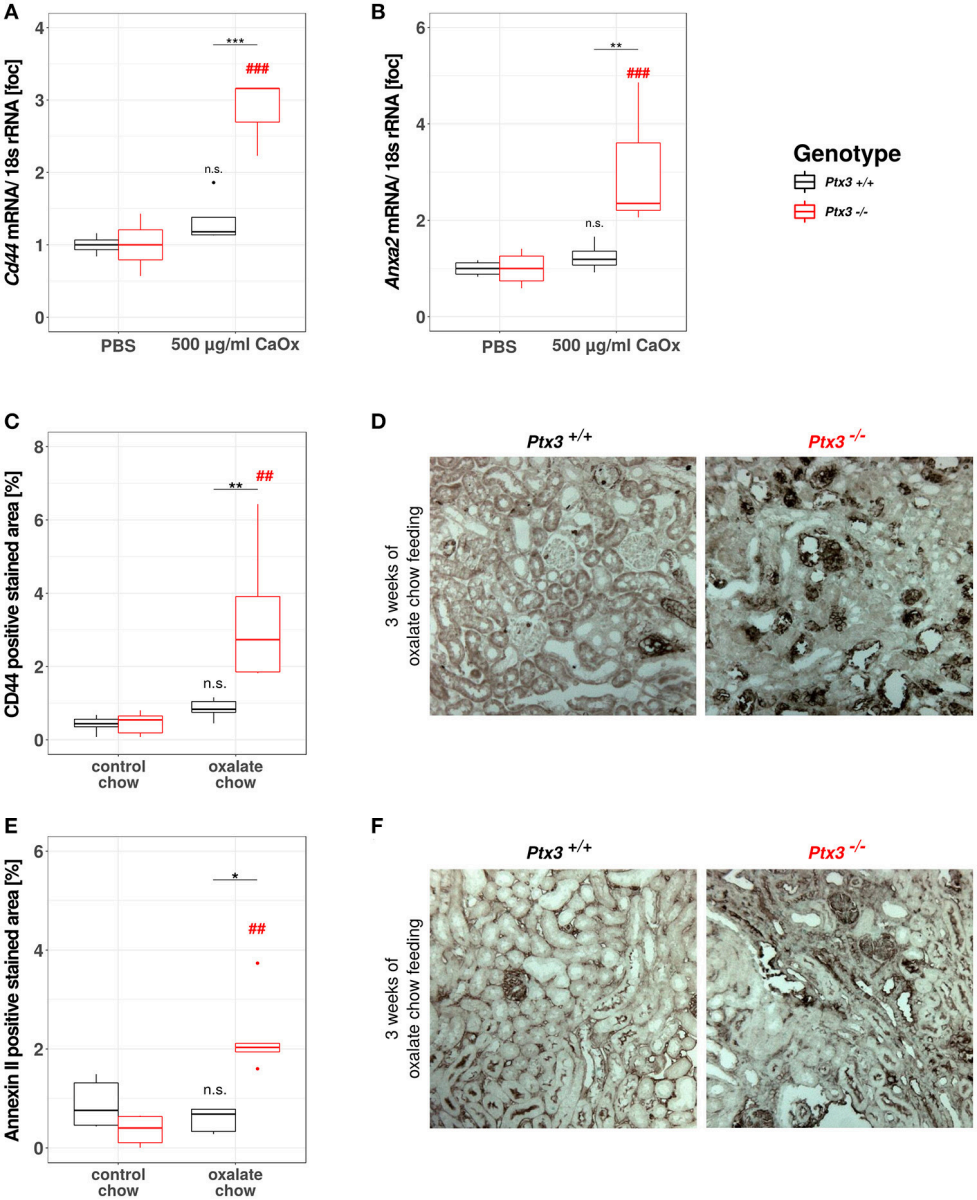
PTX3 abolishes crystal adhesion molecule expression *in vitro* and *in vivo*. **(A,B)** Primary murine proximal tubular cells were isolated from *Ptx3*^+/+^ and *Ptx3*^−/−^ littermate mice with 3 weeks of age and stimulated with PBS and 500 μg/ml CaOx crystals, respectively (*n* = 4). Total RNA was isolated and transcribed to cDNA. Subsequent qPCR for **(A)** Cd44 and **(B)** AnxaII expression was conducted. **(C–F)** *Ptx3*^+/+^ and *Ptx3*^−/−^ female littermates (10–12 weeks of age) were fed with oxalate chow for 3 weeks and formalin kidney sections were stained for **(C,D)** CD44 and **(E,F)** Annexin II, respectively (*n* = 5). n.s., not significant; *p < 0.05, **p < 0.01, ***p < 0.001 vs. wild type and respective ## and ### vs. PBS as indicated.

Based on the data gathered from our *in vitro* and *in vivo* experiments so far, we assumed that PTX3 opsonizes small CaOx crystals and thereby inhibits further crystal growth and aggregation, which would result *in vivo* in crystal plug formation, kidney injury and progressive CKD. Nevertheless, nephrocalcinosis does not solely depend on the crystal aggregation, but also on the adhesion of crystals to the luminal side of tubular cells, a process that is enabled by crystal adhesion molecules expressed on the cell surface, e.g., CD44 and annexin II. To address this point *in vitro*, we isolated primary murine proximal tubular cells from *PTX3*^+/+^ and *PTX3*^−/−^ littermates with 3 weeks of age and stimulated them with PBS or 500 μg/ml CaOx crystals (1–2 μM size). After 24 h total RNA was isolated, transcribed to cDNA and *Cd44* and *Anxa2* expression levels were quantified by qPCR (Figures 5A,B). Expression of both mRNAs was induced in tubular cells of PTX3-deficient mice stimulated with crystals only, whereas PTX3-competent cells did not upregulate the expression of either adhesion molecule. Similarly, i*n vivo*, after 3 weeks of control or oxalate chow diet, the expression of CD44 and annexin II was upregulated in tubular cells of *Ptx3*-deficient mice exposed to the oxalate chow only (Figures 5C–F). From these findings we conclude, that PTX3 not only inhibits CaOx crystal growth, but also abolishes the upregulation of crystal adhesion molecules after CaOx crystal challenge *in vitro* and *in vivo*, thereby additionally counteracting the development of nephrocalcinosis and CKD under hyperoxaluric conditions.

### Development of nephrocalcinosis and CKD under hyperoxaluric conditions is strain and sex dependent

**Figure 6:**
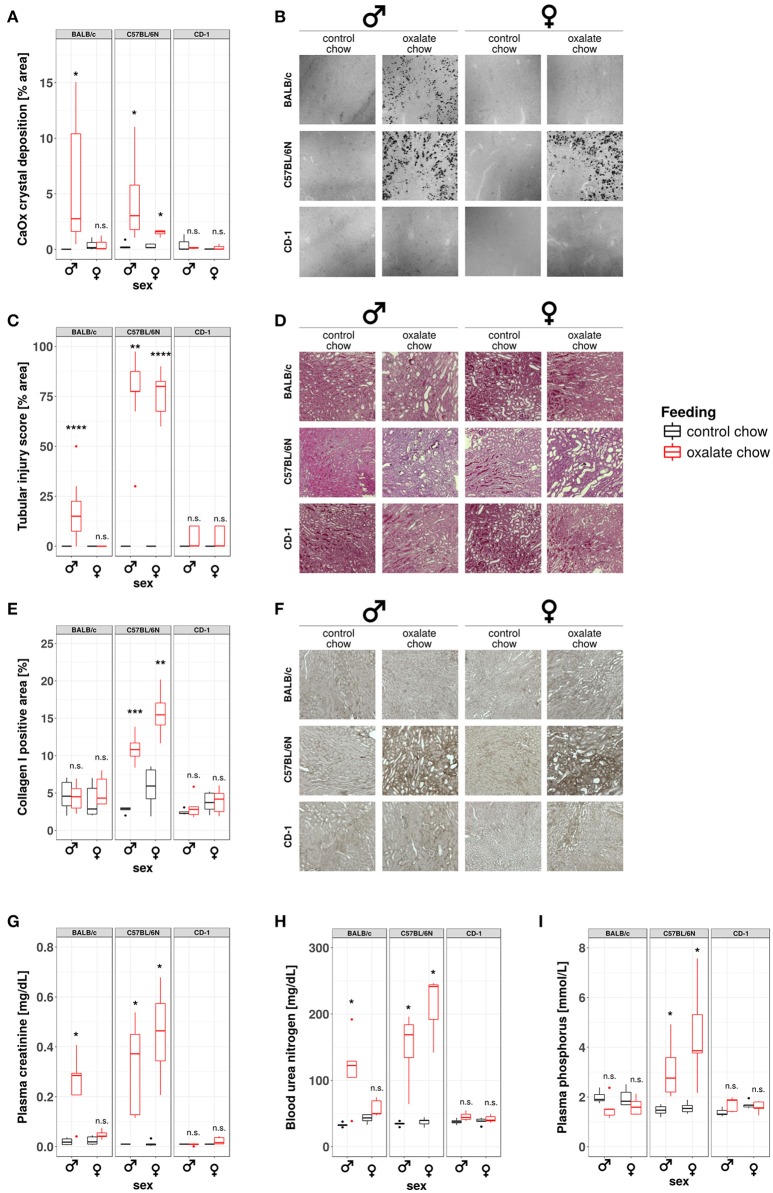
Development of nephrocalcinosis and CKD under hyperoxaluric conditions is strain and sex dependent. Male and female BALB/c, C57BL/6N, and CD-1 mice (8 weeks of age) were fed with control or oxalate chow for 3 weeks (*n* = 5). **(A)** Quantification of calcium oxalate crystal deposition was conducted using Pizzolato's method on formalin fixed kidney sections as shown in **(B)**. **(C)** Quantification of tubular injury was conducted using PAS-stained formalin fixed kidney sections as shown in **(D)**. **(E)** Quantification of Collagen I deposition was conducted using Collagen I-stained formalin fixed kidney sections as shown in **(F)**. **(G)** Plasma creatinine, **(H)** blood urea nitrogen, and **(I)** plasma phosphorus were analyzed Cobas Integra 800 autoanalyzer. n.s., not significant; *p < 0.05, **p < 0.01, ***p < 0.001, ****p < 0.0001 between treatment groups.

Our data from this and prior studies (43) suggest that the model of hyperoxaluria-induced nephrocalcinosis might be strain-dependent. To address this point, we compared the phenotypes of hyperoxaluric and control male and female mice on C57BL/6N, BALB/c, and CD-1 backgrounds, respectively. Pizzolato's staining method for CaOx crystals revealed, that out-bred CD-1 mice were not susceptible to nephrocalcinosis in our model (Figures 6A,B). In BALB/c mice, only males displayed CaOx crystal deposition, whereas females were protected. C57BL/6N mice of both sexes were susceptible to hyperoxaluria-induced nephrocalcinosis. In line with the presence of CaOx deposits in the kidneys, only BALB/c male as well as male and female C57BL/6N animals displayed tubular injury in PAS-stained sections, although the degree of injury was less profound in BALB/c compared to C57BL/6N (Figures 6C,D). Remarkably only hyperoxaluric C57BL/6N animals showed signs of fibrotic tissue remodeling, as assessed by collagen I-stained sections (Figures 6E,F), whereas BALB/c male mice did not. Characterizing the excretory kidney function we measured plasma levels of creatinine (Figure 6G), urea nitrogen (Figure 6H) and phosphorus (Figure 6I). Hyperoxaluric C57BL/6N mice of both sexes showed significantly increased levels of all three markers, whereas hyperoxaluric BALB/c male mice did not develop hyperphosphatemia and displayed lower levels of plasma creatinine and BUN compared to C57BL/6N. Based on this data we questioned whether the dependency of crystal formation from sex and strain was associated with the intrarenal levels of the PTX3 protein. Immunohistochemistry revealed a downregulation of PTX3 in male and female C57BL/6N mice fed with high oxalate diet compared to control animals, a phenomenon partly observed in male BALB/c animals also, although to a lesser extend (Supplementary Figures 1A,B). Non-crystal-forming female BALB/c and CD-1 mice of both sexes did not show PTX3 regulation. The animals with high oxalate diet which showed crystal deposition in the kidney were negative for PTX3, as shown using immunohistochemistry (Supplementary Figures 1C,D) and Western blotting in urine (Supplementary Figures 1E,F).

We conclude that genetic factors control susceptibility for hyperoxaluria-related nephrocalcinosis and that PTX3 is an endogenous inhibitory factor preventing nephrocalcinosis and its deterious consequences, e.g., by limiting crystal growth and by induction of crystal adhesion molecules.

## Discussion

We had speculated that the long pentraxin PTX3, an opsonin known to interact with dead cells and other extracellular microparticles, would affect CaOx crystal aggregation and growth, central pathomechanisms in kidney stone disease and nephrocalcinosis. Our data revealed that recombinant PTX3 limits CaOx crystal aggregation *in vitro*. Furthermore, during hyperoxaluria *in vivo* renal tubular cells secrete PTX3 which serves as an endogenous inhibitor of intrarenal crystal aggregation and adhesion, nephrocalcinosis, kidney injury, and subsequent CKD. Although not regulated in all investigated strains and sexes in the same manner, PTX3 protein levels associated with a lack of crystal formation in high oxalate diet exposed animals, further supporting a functional role of PTX3 as a calcium oxalate crystallization inhibitor. Our study identified a novel role of the opsonin and immune regulator PTX3 as a modulator of CaOx crystal aggregation and adhesion. Our data showed that PTX3 is one of several endogenous inhibitors of stone formation in nephrocalcinosis and potentially in urolithiasis or other crystallopathies.

The PTX3 protomer subunit comprises an N-terminal region, unrelated to any known protein structure, and a C-terminal domain, homologous to the short pentraxins CRP and SAP. In our study, we did not investigated which domain of the PTX3 protein mediates the inhibitory functions described above. This will be the subject of future investigations. In this regard, it is worth noting that other proteins, that are known to inhibit growth and aggregation of calcium oxalate crystals, e.g., Tamm-Horsfall glycoprotein, a_1_-microglobulin or prothrombin fragment 1 (44), do not share well-characterized homologous amino acid sequences with PTX3. In the past scientific approaches to identify proteins with function in stone formation were usually conducted by characterizing protein composition of urinary stones (45, 46). In doing so, the possible contribution of carbohydrate side chains of proteins was discussed (45). Subsequent research supported the notion that protein-beared oligosaccharides could be involved in the overall inhibitory effect of glycoproteins on crystal growth, e.g., sialic acid side chains on Tamm-Horsfall protein (47, 48). The most efficient modifiers of calcium stones have a large number of carboxylic, hydroxyl, sulfate or phosphate functions on their structure (49). The PTX3 protein has a single N-glycosylation site in the C-terminal domain that is occupied by fucosylated and sialylated complex type sugars (37, 38). This post-translational modification has been documented to affect the binding of PTX3 to a number of ligands, including complement components (37, 50) and P-selectin (51), thereby effectively altering the immune-modulatory properties of PTX3. It is worth noting here that throughout our study a recombinant preparation of PTX3 from Per.C6 cells was used, which is N-linked glycosylated (based on SDS-PAGE of the PNGase F-treated protein; data not shown). However, we cannot rule out that the glycosidic moiety of the Per.C6-derived protein might differ from that of the CHO-expressed PTX3 (i.e., that was used in previous reports). Although beyond the scopes of the study, this is an interesting point that deserves further investigations. Nonetheless, based on the body of literature available, it appears licit to speculate that the glycosylation status of PTX3 (with major regard to fucose and sialic acid-content) might influence the protein's properties as an inhibitor of crystal growth.

Another possible route of action for PTX3 that needs further investigation, is related to its association with TNF-α signaling. We were previously able to show, that crystal adhesion to the luminal membrane of renal tubules requires TNFR signaling (28). The connection between TNF-α and PTX3 is well-established and indicates that PTX3 expression is a downstream event of TNF-α signaling (52), but also that PTX3 itself can inhibit TNF-α induced transcription factor activation (53). Hence, altered TNFR signaling might be involved in the phenotype observed in *Ptx3*-deficient mice.

Although technically feasible, we did not consider to recover the wild type mouse nephropathy phenotype by reconstituting the lack of PTX3 in *Ptx3*-deficient mice by injecting recombinant PTX3 because the PTX3 protein octamer is too large to pass the glomerular filter (40). Without a chance of reaching the tubular lumen, exogenous administered PTX3 cannot exert protective effects under hyperoxaluric conditions, which greatly limits its potential usefulness as a therapeutic intervention.

Nevertheless, we identified PTX3 as a previously unknown endogenous protein inhibitor of intrarenal CaOx crystal growth and adhesion (49). The field agrees that nephrocalcinosis, kidney stone disease and urolithiasis do not have a monogenic cause (54). Rather an increasing list of candidate genes is assembled by whole genome genotyping approaches using nephrolithiasis patient cohorts (55, 56). Our data suggest to include *Ptx3* in the list of candidate genes, that might contribute to a better understanding of stone diseases and prove useful in future patient diagnosis and management. Additionally, the investigation of different strains and sexes revealed, that decreased PTX3 protein expression associates with stone formation in some animals (C57BL/6N male and female), whereas other animals, that are not susceptible for hyperoxaluria-induced nephrocalcinosis, did either not show any PTX3 protein regulation (CD-1 male and female) or upregulated PTX3 under hyperoxaluric conditions (*Ptx3-*competent mice of the mixed B6;129 background). These *in vivo* observations might serve as a hint for the clinical setting, where haplotype compositions of each patient differ and some stone formers might show differences in PTX3 protein regulation, whereas others do not. PTX3 polymorphisms have already been shown to greatly influence the susceptibility of patients to aspergillosis, probably due to mRNA instability in some SNPs (57). Our data provide the rational for a similar study regarding kidney stone patients.

We reported the model of experimental hyperoxaluria-induced nephrocalcinosis to be valuable and clinically relevant for CKD research in male and female C57BL/6N mice (43). Next, the absence of nephrocalcinosis in *Ptx3*^+/+^ animals on B6;129 background 3 weeks after model induction prompted us to study issues of strain and sex dependency in this model. With both sex and background affecting CaOx deposition with different degrees we believe that this model became even more valuable for studying mechanisms and risk factors related to kidney stone diseases. *In silico* comparison and transcriptome analysis of different murine strains and sexes available are very likely to draw a very precise picture on disease related genetic risk factors and possible therapeutic targets.

For CKD research—apart from the unique pathomechanisms only found in crystallopathies—strain and sex dependency of the model point out that researchers need to apply great care when using genetically-modified organisms, that were generated from different backgrounds or not completely backcrossed. Also, this does not only hold true for hyperoxaluria-induced nephrocalcinosis, but for a variety of other renal inflammatory models and conditions, e.g., thioglycolate-induced peritonitis (58), inflammatory response and albuminuria in response to albumin-overload (59), anti-GBM glumerulonephritis (60) as well as response to nephrotoxins (61).

In summary, the opsonin and immune regulator PTX3 also modulates CaOx crystal aggregation and adhesion to tubular cell membranes and is therefore one of several endogenous inhibitors of stone formation in nephrocalcinosis and potentially in urolithiasis or other crystallopathies.

## Materials and methods

### Animal studies

BALB/c, C57BL/6N, and CD-1 mice were purchased from Charles River Laboratories (Sulzfeld, Germany). *Ptx3-*deficient mice in the B6;129 background were obtained from Alberto Mantovani/ Charles River Italy and were generated as previously described (62). Mice were co-housed in groups of five in filter top cages with unlimited access to food and water. Cages, nestlets, food, and water were sterilized by autoclaving before use. Eight-to-twelve-weeks-old male mice were used for experiments. Oxalate diet was prepared by adding 50 μmol/g sodium oxalate to a calcium-free standard diet (Ssniff, Soest, Germany) as previously described (43, 63). Mice were sacrificed at day 7, 14, and 21 after starting oxalate diet. Plasma and urine samples were collected and GFR was measured at different time points before cervical dislocation. Urine was immediately acidified for oxalate estimations or centrifuged for 10 min at 10,000 g for cellular component removal and stored at −80°C. One part of each kidney was fixed in formalin and subsequently embedded in paraffin for histological analysis. All experimental procedures were approved by the local government authorities.

### Assessment of renal injury

Kidney sections of 2 μm were stained with periodic acid-Schiff (PAS) reagent, and the tubular injury was scored by assessing the percentage of necrotic tubules and presence of tubular casts. Pizzolato's staining was used to visualize CaOx crystals and crystal deposit formation in the kidney was evaluated as described (43). Fibrotic areas were identified by immunostaining for αSMA (Dako GmbH, Germany) and collagen I (Abcam, Cambridge, UK). The expression of crystal adhesion molecules e.g., CD44 and Annexin II was identified by immunostaining for CD44 and Annexin II (both Abcam, Cambridge, UK). PTX3 expression was identified in paraformaldehyde-fixated and cryo-cut tissue samples using a polyclonal rabbit anti-human PTX3 antibody (Enzo Life Sciences, Farmingdale, USA).

Quantification of Pizzolato's silver stain and immunostainings was done using Image J software. All assessments were performed by an observer blinded to the experimental condition. Plasma BUN, creatinine and phosphorus levels were measured using a Cobas Integra 800 autoanalyzer (Roche, Mannheim, Germany).

Assessing the signal intensity of the PTX3 staining in C57BL/6N, BALB/c, and CD-1 animals was not feasible using ImageJ, as big calcium oxalate crystal deposits were visible in the cryosections, showing the same level of contrast as the immunostaining. Hence, PTX3 staining was semi-quantitatively assessed using a scoring system where a strong positive signal was scored with a value of 2, a weak signal with a value of 1 and absence of signal with a value of 0. This evaluation was conducted independently for the cortex, medulla and papilla of one kidney section and the overall score resulted from the sum of all values, possibly raging of 0 to 6.

### Transcutaneous measurement of glomerular filtration rate (GFR) in conscious mice

For GFR measurement mice were anesthetized with isoflurane and a miniaturized imager device built from two light-emitting diodes, a photodiode and a battery (MediBeacon, Mannheim, Germany) was mounted via a double-sided adhesive tape onto the shaved animals' neck (64). For the duration of recording (~1.5 h) each animal was conscious and kept in a single cage. Prior to the intravenous injection of 150 mg/kg FITC-sinistrin (MediBeacon, Mannheim, Germany), the skin's background signal was recorded for 5 min. After removing the imager device the data were analyzed using MPD Lab software (MediBeacon, Mannheim, Germany). The GFR [μl/min] was calculated from the decrease of fluorescence intensity over time (i.e., plasma half-life of FITC-sinistrin) using a two-compartment model, the animals body weight and an empirical conversion factor (64).

### X-ray diffraction analysis of intrarenal crystals

The analysis of the freeze dryed mouse kidney tissue by X-ray powder diffraction was performed with an Empyrean setup from PANalytical. A Cu x-ray tube (line source of 12 × 0.04 mm^2^) provided CuKa radiation with l = 0.1542 nm. The Kb line was removed by a Ni filter. Source and detector moved in the vertical direction around a fixed horizontal sample. After passing a divergence slit of 1/8° and an anti-scatter slit of 1/4°, the beam reached the sample at the center of a phi-chi-z stage. In the Bragg-Bretano geometry used, the beam was refocused at a secondary divergence slit of 1/4°. Finally, the signal was recorded by a pixel detector (256 × 256 pixels of 55 μm) as a function of the scattering angle 2θ. Subsequently, the peak positions were calculated from *q* = 2π/*d* = (4π/λ) sinθ, in which **q** is the scattering vector. The detector was used in a scanning geometry that allowed all rows to be used simultaneously.

### Flow cytometry

Whole kidneys were chopped and digested for 30 min at 37°C using a collagenase and DNase I solution (both Sigma-Aldrich, Taufkirchen, Germany). Samples were further strained through a 70 μm mesh and washed with PBS. Dead cells were excluded from analysis using the Zombie NIR™ Fixable Viability Kit according to the manufacturer's instructions and as described before (65). CD45^+^ positive cells were identified using a PE-Cy™5 labeled antibody (clone 30-F11, BD Biosciences, Franklin Lakes, USA). Analysis was conducted using FlowJo v10.0.

### Cell culture studies

Primary tubular epithelial cells (pTECs) were isolated from kidneys of mice with 3 weeks of age and were maintained in DMEM/F12 containing 10% fetal calf serum, 1% penicillin–streptomycin, 125 ng/ml prostaglandin E1 (Calbiochem, Darmstadt, Germany), 25 ng/ml EGF, 1.8 μg/ml l-thyroxine, 3.38 ng/ml hydrocortisone and 2.5 mg/ml of insulin-transferrin-sodium selenite supplement (I-T-SS) (all from Sigma-Aldrich, Taufkirchen, Germany unless mentioned) as previously described (66, 67). All cells were cultured in an incubator at 37°C, 5%CO_2_, and stimulated with crystals of CaOx (1–2 μm size; Alfa Aesar, Karlsruhe, Germany).

### RNA preparation and real-time quantitative PCR

Total RNA was isolated from pTECs using a Qiagen RNA extraction kit (Düsseldorf, Germany) following the manufacturer's instructions. After quantification RNA quality was assessed using agarose gels. From isolated RNA, cDNA was prepared using reverse transcriptase (Superscript II; Invitrogen, Carlsbad, USA). Real-time quantitative PCR (qPCR) was performed using SYBRGreen PCR master mix and was analyzed with a Light Cycler 480 (Roche, Mannheim, Germany). All gene expression values were normalized using 18S rRNA as a housekeeping gene. All primers used for amplification were from Metabion (Martinsried, Germany), and are listed in Table 1.

**Table 1:** Primer sequences for qPCR.

**Target**		**Primer sequence**
CD44	Forward Reverse	5′-AGCGGCAGGTTACATTCAAA-3′ 5′-CAAGTTTTGGTGGCACACAG-3′
Annexin II	Forward Reverse	5′-GCACATTGCTGCGGTTTGTCAG-3′ 5′-CACCAACTTCGATGCTGAGAGG-3′
18s RNA	Forward Reverse	5′-GCAATTATTCCCCATGAACG-3′ 5′-AGGGCCTCACTAAACCATCC-3′

### Immunoblotting

After determination of protein concentrations in urine samples, 50 μg of the protein solution was mixed with 4× sodium dodecyl sulfate loading buffer and was denatured at 95°C for 5 min for western blot analysis. Proteins were then separated by sodium dodecyl sulfate-polyacrylamide gel electrophoresis and transferred to a polyvinylidene difluoride membrane. Non-specific binding to the membrane was blocked for 1 h at room temperature with 5% non-fat milk in tris-buffered saline. The membranes were then incubated overnight at 4°C with a primary antibody for PTX3 (clone 2C3, Hycult Biotechs, Plymouth Meeting, USA) followed by incubation with a secondary rabbit anti-mouse IgG HRP-labeled antibody. Immunostained bands were detected using a chemiluminescence kit (ECL kit, GE Healthcare, Cardiff, UK). Equal loading of urinary proteins was visualized by Ponceau Red staining (1 h at RT). Stain intensities for both stainings were further analyzed by densitometry (Image J). Murine PTX3 from urine samples as well as the recombinant PTX3 positive control showed the expected band around 45 kDa.

### *In Chemico* generation and characterization of calcium oxalate crystals

Calcium oxalate crystals of defined size and shape were generated as described elsewhere (68). In short, 10 mM sodium oxalate and 1 mM calcium chloride solutions were prepared using a 10 mM TRIS-HCl buffer (pH 7.3). For CaOx crystal generation 1 volume sodium oxalate solution was incubated over night at 4°C with 0.5 volume of either PBS or rhPTX3 in different concentrations. Next, 1 volume calcium chloride solution was added and kept at 4°C for 24 h. CaOx crystal size in the different preparations was assessed using bright light microscopy or flow cytometry.

### Recombinant human PTX3

Human PTX3 was recombinantly expressed in a PerC6 human cell line. Briefly, Per.C6 cells (Crucell, Leiden, The Netherlands) were stably transfected with a plasmid vector (pcDNA2001Neo-hPTX3) carrying the human PTX3 cDNA under control of the CMV promoter. After transfection, a highly producing clone was selected and expanded for protein expression. The recombinant protein was purified from conditioned medium using a multi-step chromatography strategy as previously described (69). Protein homogeneity in the end-product was assessed by analytical size exclusion chromatography (SEC), SDS-PAGE and immunoblotting (39). N-linked glycosylation of the purified protein was investigated using PNGase F (Sigma Aldrich) as previously described (37).

### Statistical analysis

Data are presented as mean with SEM or as boxplot statistics. Prior to every other statistical analysis, data were checked for normal distribution (Shapiro-Wilk test), homoscedasticity (Levene's test) and outliers (Grubb's test). Normally distributed and homoscedastic data sets were tested for statistical significant differences via ANOVA and *post-hoc* Bonferroni's correction was used for multiple comparisons. Heteroscedastic data were corrected following Games-Howell's *post-hoc* test. Not normally distributed data sets were compared using Kruskal-Wallis and Nemenyi testing. A value of *p* < 0.05 was considered to indicate statistical significance. *P*-values were indicated as *p* > 0.05 n.s., *p* < 0.05 ^*^ (or #), *p* < 0.01 ^**^ (or ##), *p* < 0.001 ^***^ (or ###).

## Ethics statement

All experimental procedures were carried out according to the German Animal Care and Ethics legislation and were approved by the local governmental authorities, i.e., the Ethical Committee of the Regierung von Oberbayern, permit no. (AZ) 55.2-1-54-2532-63-12 and 55.2-1-54-2532-143-13.

## Author contributions

JM: design and conduction of experiments, data analysis, manuscript preparation; SM: design and conduction of experiments, data analysis; JM and SM contributed equally to the project; SS: flow cytometry conduction and analysis; LA: western blot conduction and analysis; ZZ: immunhistochemistry analysis; PB: X-ray diffraction conduction and analysis, manuscript preparation; KR: X-ray diffraction conduction and analysis; AI: provision of recombinant protein, experimental design, and manuscript preparation; CG: provision of transgenic animals, recombinant protein, PTX3 immunohistochemistry; AM: provision of transgenic animals, recombinant protein, PTX3 immunohistochemistry; H-JA: experimental design, data analysis, funding, manuscript preparation.

### Conflict of interest statement

The authors declare that the research was conducted in the absence of any commercial or financial relationships that could be construed as a potential conflict of interest.

## Supplementary material

Supplementary Figure 1 PTX3 protein expression under hyperoxaluric conditions in different strains and sexes. Male and female BALB/c, C57BL/6N, and CD-1 mice (8 weeks of age) were fed with control or oxalate chow for 3 weeks (*n* = 5). **(A)** Scoring PTX3 positivity was performed on PTX3 immunostained cryosections as shown in **(B)**. Calcium oxalate crystal deposits are indicated with asterisks. **(C–F)** The figures include only those animals from the study, that were exposed to oxalate diet, not the control animals. **(C)** Comparing PTX3 staining scores between animals developing crystal deposition and those that did not, based on PTX3 immunohistochemistry on cryosections and Pizzolato's staining for oxalate crystals on paraffin sections **(D)**. **(E)** Comparing optical densities of immunoblots for PTX3 from urine samples (25 μg protein/lane) analogous to **(C,D)**. **(F)** Western blot for PTX3 (~45 kDa, upper panel) and Ponceau Red staining for major urinary proteins (mainly a2u-globulins, ~20 kDa, lower panel) as a loading control. Data are from one experiment. n.s., not significant; ^*^*p* < 0.05, ^**^*p* < 0.01, ^****^*p* < 0.0001 between groups as indicated.
