# Supplementary figures and images for: The Long Pentraxin PTX3 Is an Endogenous Inhibitor of Hyperoxaluria-Related Nephrocalcinosis and Chronic Kidney Disease
Source: Front Immunol. 2018 Sep 25;9:2173. doi: 10.3389/fimmu.2018.02173 (PMC6167460; doi:10.3389/fimmu.2018.02173)

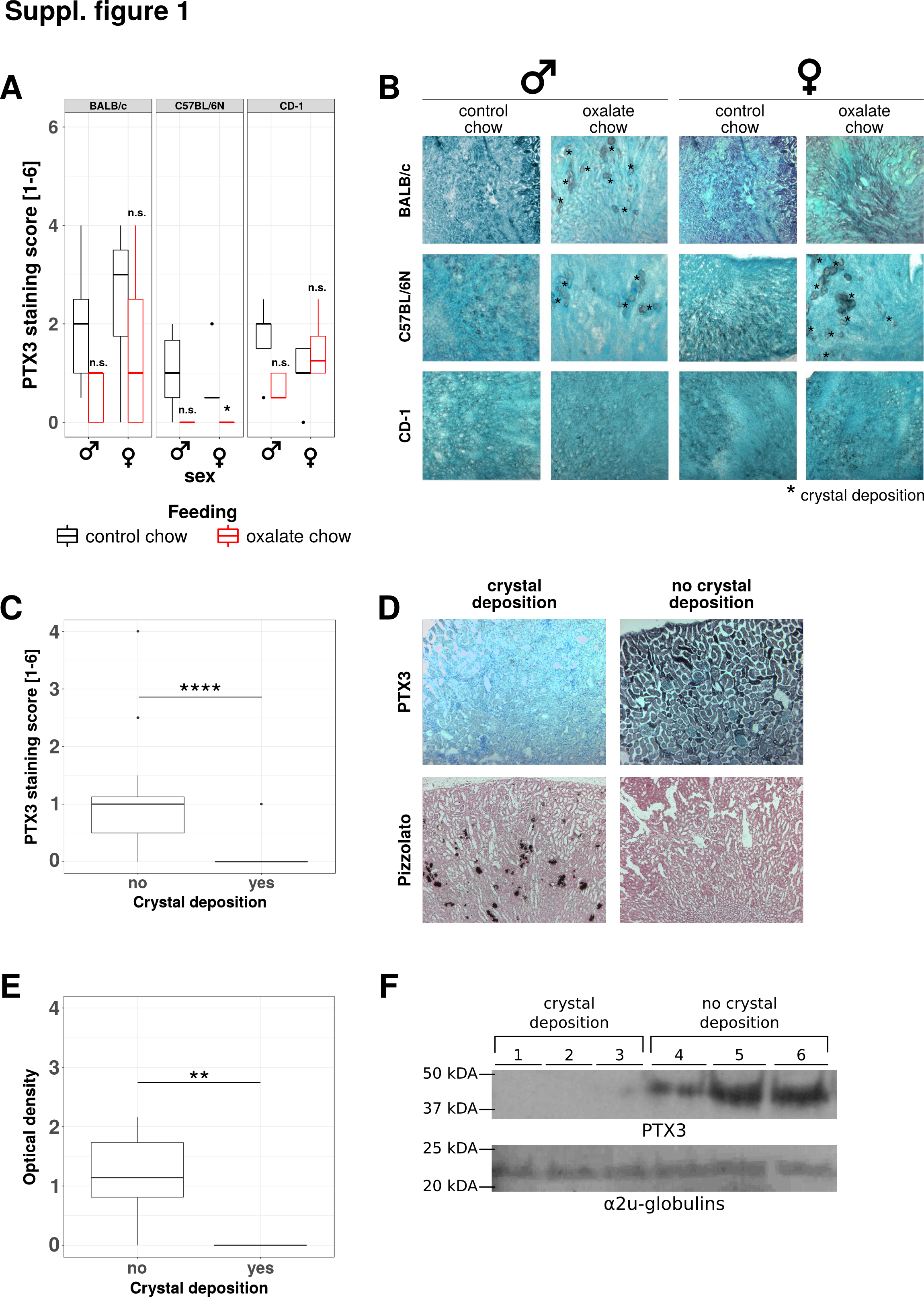

Supplement: Supplementary Figure 1 — PTX3 protein expression under hyperoxaluric conditions in different strains and sexes. Male and female BALB/c, C57BL/6N, and CD-1 mice (8 weeks of age) were fed with control or oxalate chow for 3 weeks (n = 5). (A) Scoring PTX3 positivity was performed on PTX3 immunostained cryosections as shown in (B). Calcium oxalate crystal deposits are indicated with asterisks. (C–F) The figures include only those animals from the study, that were exposed to oxalate diet, not the control animals. (C) Comparing PTX3 staining scores between animals developing crystal deposition and those that did not, based on PTX3 immunohistochemistry on cryosections and Pizzolato's staining for oxalate crystals on paraffin sections (D). (E) Comparing optical densities of immunoblots for PTX3 from urine samples (25 μg protein/lane) analogous to (C,D). (F) Western blot for PTX3 (~45 kDa, upper panel) and Ponceau Red staining for major urinary proteins (mainly a2u-globulins, ~20 kDa, lower panel) as a loading control. Data are from one experiment. n.s., not significant; *p < 0.05, **p < 0.01, ****p < 0.0001 between groups as indicated. [file Image_1.JPEG]
